# First-Principles Study of Cu-Based Inorganic Hole Transport Materials for Solar Cell Applications

**DOI:** 10.3390/ma15165703

**Published:** 2022-08-18

**Authors:** Adriana Pecoraro, Pasqualino Maddalena, Michele Pavone, Ana B. Muñoz García

**Affiliations:** 1 Department of Physics “Ettore Pancini”, University of Naples Federico II, 80126 Napoli, Italy; 2 Department of Chemical Sciences, University of Naples Federico II, 80126 Napoli, Italy

**Keywords:** perovskite solar cells, dye sensitized solar cells, copper-based hole transport materials

## Abstract

Perovskite solar cells (PSCs) and dye-sensitized solar cells (DSCs) both represent promising strategies for the sustainable conversion of sunlight into electricity and fuels. However, a few flaws of current devices hinder the large-scale establishment of such technologies. On one hand, PSCs suffer from instabilities and undesired phenomena mostly linked to the perovskite/hole transport layer (HTL) interface. Most of the currently employed organic HTL (e.g., Spiro-OMeTAD) are supposed to contribute to the perovskite decomposition and to be responsible for charge recombination processes and polarization barriers. On the other hand, power conversion efficiencies (PCEs) of DSCs are still too low to compete with other conversion technologies. Tandem cells are built by assembling p-type and n-type DSCs in a cascade architecture and, since each dye absorbs on a different portion of the solar spectrum, the harvesting window is increased and the theoretical efficiency limit for a single chromophore (i.e., the Shockley–Queisser limit) is overcome. However, such a strategy is hindered by the lack of a p-type semiconductor with optimal photocathode features. Nickel oxide has been, by far, the first-choice inorganic p-type semiconductor for both PV technologies, but its toxicity and non-optimal features (e.g., too low open circuit voltage and the presence of trap states) call for alternatives. Herein, we study of three p-type semiconductors as possible alternative to NiO, namely CuI, CuSCN and Cu_2_O. To this aim, we compare the structural and electronic features of the three materials by means of a unified theoretical approach based on the state-of-the art density functional theory (DFT). We focus on the calculation of their valence band edge energies and compare such values with those of two widely employed photo-absorbers, i.e., methylammonium lead iodide (MAPI) and the triple cation MAFACsPbBrI in PSCs and P1 and Y123 dyes in DSCs, given that the band alignment and the energy offset are crucial for the charge transport at the interfaces and have direct implications on the final efficiency. We dissect the effect a copper vacancy (i.e., intrinsic p-type doping) on the alignment pattern and rationalize it from both a structural and an electronic perspective. Our data show how defects can represent a crucial degree of freedom to control the driving force for hole injection in these devices.

## 1. Introduction

World energy consumption and environmental concerns call for the replacement of fossil fuels with renewable sources. To this end, sunlight is an extremely powerful energy source and has the greatest potential, such that the scientific community is hastening toward mass production of photovoltaic (PV) devices to drive this energy transition. Besides presenting high conversion efficiencies and long-term stability, PV devices for commercialization should be cost-effective, i.e., make use of abundant materials and facile fabrication setups, and environmentally friendly, i.e., without toxic elements.

Different technological approaches able to convert sunlight into electricity or fuels have succeeded over the years. Silicon-based devices, the most evolved and mature technology, dominate the solar industry, but two related yet different technologies have seized the emerging PV field due to their tunability and versatility: dye-sensitized solar cells (DSCs), pioneered in 1991 by Grätzel [1], and perovskite solar cells (PSCs) [2], first described in 2009.

**Figure 1:**
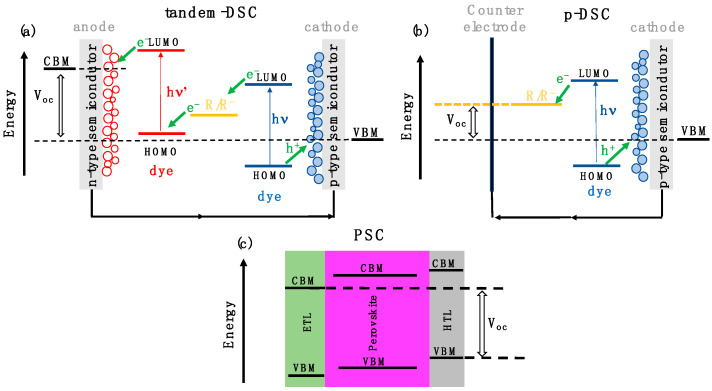
Energy levels alignment required for efficiently working p-type DSC (**a**), tandem-DSC (**b**) and PSC (**c**).

DSCs’ competitiveness on the market stems from the affordable costs, simple preparation methods, lightweight construction and superior suitability for indoor use thanks to their strong performances at diffuse and low-intensity light [3]. In traditional n-type DSCs (Grätzel cells), photon-to-electron conversion is triggered by organic dyes that, after light absorption and charge separation, inject free electrons to the mesoporous n-type semiconductor layer (typically TiO_2_) where they are anchored. Dye regeneration is ensured by a charge transport material, usually a liquid electrolyte, that closes the circuit. To date, the highest power conversion efficiency (PCE) rates have been achieved at around 13–14% [4,5], meaning that DSCs still fall behind their competitors. A strategy to overcome the intrinsic theoretical efficiency limits for a single chromophore (i.e., the Shockley–Queisser limit) consists of coupling p- and n-type DSCs in a configuration fulfilling the band energy alignment shown in Figure 1a. While in a n-DSC, the electron is transferred from the LUMO of the excited dye to the anode conduction band, in a p-DSC, the hole is injected from the HOMO of the excited dye to the valence band maximum (VBM) of the cathode. For such a hole transfer to occur, the energy levels of the involved components should fulfill the scheme sketched in Figure 1b, i.e., the VBM of the semiconductor should be higher than the dye HOMO. This cascade configuration is known as a tandem cell [6] and is able to widen the solar spectrum portion accessible to the cell thanks to the presence of two dyes with optimally chosen bandgap energies, matched with chemically and electronically suitable p- and n-type semiconductors. In spite of great research efforts devoted to tandem devices, their development is hindered mainly by the lack of a p-type photoelectrode with optimal features. NiO is the most commonly employed photocathode in p-DSCs [3], but besides some concerns about its toxicity, several drawbacks, such as low electrical conductivity, low hole mobility and high valence band edge potential with respect to the most common I^−^/I^−^_3_ electrolyte, result in too low PCEs of NiO-based DSCs [7]. The non- competitive state-of-the-art PCE and the instabilities related to the electrode corrosion held by the use of liquid electrolyte are the main challenges in upscaling their manufacture.

On the other hand, PSCs have spurred enormous interest in the last few years due to their rapidly increasing PCEs, which reach 26% [8]. They first appeared in 2009 [9] when a hybrid organic–inorganic perovskite, the archetypal methyl-ammonium lead iodide (MAPI), was used to substitute the organic dye sensitizer in a DSC and reached a 3.8% PCE. However, such a device was not stable since the liquid electrolyte rapidly dissolved the perovskite. PCEs as high as 9% were obtained when Spiro-OMeTAD, as a solid-state hole transport layer (HTL), was substituted as the liquid electrolyte [10]. Despite growing efficiencies achieved with MAPI derivatives, the critical hurdles to practical application are certainly severe stability issues that hinder large-scale market distribution of PSCs. In particular, thermal treatment, humidity and light illuminations are instability sources affecting mainly the chemically active charge transport layers and degradation occurring at interfaces. On one hand, TiO_2_ is the most widely used electron transport material (ETL) in high-performing devices, however it suffers from degradation under UV irradiation. On the other hand, Spiro-OMeTAD, together with other organic HTLs, such as polystyrene sulfonate (PEDOT: PSS) and PTAA, still remain the materials of choice as hole transport compounds but bring about some concerns about the device stability. The structures of these compounds are reported in Figure S1 of the Supporting Information. These materials are indeed able to provide high open circuit potentials (V_oc_) and high PCEs, but they exhibit scarce conductivity and hole mobility, and the doping strategies adopted to increase them lead to thermal instability, which is a major bottleneck for PSC commercial viability [11,12]. NiO has been extensively used as an inorganic alternative HTL [13], however it is found to generate trap states and poor interface charge dynamics in PSCs [14]. Alternative HTLs have to be found that, as for DSCs, should possess energy levels suitably matched with the other components of the cell to allow for the charge transport across the different junctions. A schematic diagram of the desirable band alignment in PSCs is shown in Figure 1c. The VBM of the HTL should be higher in energy than the Perovskite VBM in order to provide the driving force for hole injection.

Development of suitable p-type hole-extracting materials is, thus, essential to reach a turning point on these third-generation PV devices, which could enable the development of DSC tandem cells on one hand and improve the stability of PSC devices on the other hand.

A promising alternative to NiO is represented by p-type semiconductors based on copper, which have received a great deal of attention, thanks to their chemical stability, high carrier mobility, facile synthesis [15] and excellent opto-electronic features suited for a variety of photo-electrochemical applications, including photocatalysis [16,17].

To date, devices built with these promising Cu-based inorganic materials have not yet achieved the performances delivered by other state-of-the-art HTMs due to the difficulty of finding a deposition method without side effects on the perovskite. In the conventional n-i-p configuration of PSC, they are directly deposited on the perovskite film and the chemical approaches used degrade the perovskite or lead to the formation of small grains in the material, which increase hole–electron recombination [18,19,20]. Even if this problem is absent in the inverted p-i-n architecture, where the order of materials is reversed, assembling and deposition techniques strongly impact on the quality of the contact, the presence of defects and, thus, on the performance of the cell.

In particular, Cu vacancies are responsible for the p-type behavior and have a beneficial role in enhancing p-type conductivity. However, these intrinsic defects modify the electronic properties and can lead to surface trap states or can change the matching energy levels [21].

In previous works, we have focused on Cu-based delafossites as potential alternatives to NiO for DSCs, assessing the role of doping and the surface properties [22], together with the interaction with the dye [23]. Herein, we focus on three Cu-based semiconductors, namely copper iodide (CuI), copper thiocyanate (CuSCN) and cuprous oxide (Cu_2_O). These materials are solution processable and show a proper band alignment with selected dyes and perovskites, as demonstrated by experimental studies reporting remarkably improved PCEs in both DSCs [24,25,26] and PSCs [27,28,29]. From the theoretical perspective, several studies have investigated the electronic properties of these compounds within DFT-based calculations. Odeke et al. carried out a DFT study on the electronic structure of copper thiocyanate at DFT-PBE level of theory [30], while Nolan and Elliot studied the connection between Cu defects and p-type behavior in Cu_2_O. A similar investigation correlated the hole effective mass to the presence of Cu vacancies in CuI [31]. Besides these studies that focused on the isolated compounds, a few theoretical investigations of Cu-based compounds/perovskite interfaces are also present in literature. Castellanos-Águila et al. considered the (001) MAPI/Cu_2_O interface, finding a beneficial role of Cu vacancy in PV performances [32]. Welch et al. assessed both the stability and band alignment of the fully inorganic CuI/CsPbBr_3_ interface [33], while Sajjad et al. [34] investigated the CuSCN/MAPI interface, finding a favorable type II band alignment. All these studies provide valuable insights on the applicability of these materials in PV devices and highlight some of their strength points. To the best of our knowledge, there are no works in literature that directly relate the presence of copper vacancies in these compounds to the band alignment with perovskites and dyes. We aim herein to fill this knowledge gap by performing a theoretical first-principles periodic DFT+U study to assess how the same point defect, a copper vacancy, affects the three different HTMs. In order to make a sound comparison, we describe the three materials with a similar and consistent computational prescription and discuss the possible implications on DSC and PSC performances.

For this purpose, we evaluate the bulk properties, such as lattice constants, and electronic structures confirming the well-known role of Cu vacancies in providing the p-type features to the electronic behavior. We also investigate the surface properties of the most stable facets and evaluate the valence band edge positions of all the materials through a well-established theoretical approach [35] based on the surface-slab model. We consider the presence of a copper vacancy in the inner part of each slab and dissect its role in modifying the electronic properties, rationalizing such modifications in terms of the structural changes. Besides comparing the three materials, we assess their applicability in devices involving some of the most employed photoactive materials, namely the MAPI, the triple cation MAFACsPbBrI and the P1, Y123 dyes [36]. Our first-principles analysis provides a direct and consistent comparison among the three materials for application as photocathodes/HTLs, thus deriving new insights that will help their applications in new, better performing PV devices.

## 2. Computational Details

All our calculations have been performed within the framework of periodic density functional theory (DFT) with projector-augmented wave (PAW) potentials [37,38] and a plane wave (PW) basis set with the Vienna ab Initio Simulation Package (VASP, version 5.4.4) code [39,40,41,42].

We have exploited the Perdew, Burke and Ernzerhof (PBE) approximation for the exchange and correlation density function [43]. In order to amend the self-interaction error associated to strongly correlated Cu *d* electrons, we have applied the rotationally invariant DFT+U approach of Dudarev [44], as implemented in VASP [45]. Following previous works on Cu(I)-containing oxides, we have set a U-J (Cu) value equal to 6.0 eV [22,23,46,47]. Dispersion interactions have been treated within the Grimme’s D3 framework with the damping scheme proposed by Becke and Johnson (D3-BJ) [48,49,50]. We have set a SCF energy threshold of 10^−5^ eV and a total force threshold of 0.03 eV/Å for geometry optimizations.

The plane wave energy cutoff has been set to 700 eV in all calculations. Ґ-Centered k-point sampling grids of 4 × 4 × 4, 6 × 6 × 2 and 6 × 6 × 6 have been used for CuI, hexagonal CuSCN and Cu_2_O bulk materials, respectively. The sampling of reciprocal space for the surface slabs has been scaled accordingly. All atoms of bulks and surface slabs have been freely relaxed without symmetry constraints during optimizations.

## 3. Results and Discussion

Firstly, we characterized the minimum energy bulk structures for the three materials under study. We considered the most stable bulk phases for each pristine compound and suitable supercells when the copper defects have been introduced. In particular, among all CuI polymorphs, we examined the γ-phase [51] (*F-43m* space group), which is stable within the expected solar cell operating temperature range. The p-type CuI has been modeled by removing one Cu atom from the 64-atom 2 × 2 × 2 supercell for a Cu/I ratio of 0.97. This supercell size ensures non-interacting vacancy images as separated by about 12 Å. Regarding CuSCN, we selected the hexagonal β-phase [52] (*P6_3_mc*) because it is more frequent than the orthorhombic α-phase [53] (*Pbca* group). In this case, we performed our calculations on a 92-atom pseudo-cubic cell obtained by orthogonalizing the 4 × 3 × 1 hexagonal supercell. For modeling p-CuSCN, we eliminated two copper atoms at a distance of about 10 Å in order to obtain a Cu/SCN ratio of 0.92, which lies in the range where the best PV performances have been registered (0.92–0.96) [54]. Cu_2_O crystallizes in a cubic structure (*Pm-3m* group) [55]. Here, we mimicked the presence of vacancies by removing one copper atom form the 2 × 2 × 2 40-atom supercell, which resulted in a Cu/O ratio of 1.94. Such bulk structure models are reported in Figure S2 of the Supporting Information, while the theoretically obtained lattice constants, in comparison to experimental values from literature, are reported in Table S1.

**Figure 2:**
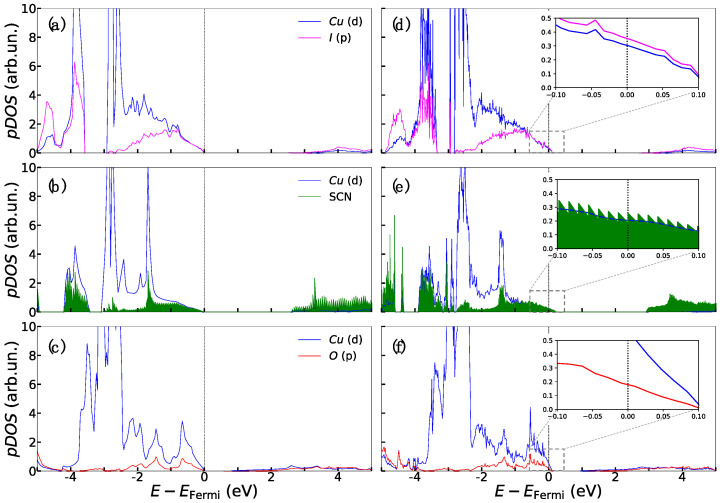
Atom- and orbital angular momentum-projected density of states (pDOS) for the bulk structures of CuI (**a**,**d**), CuSCN (**b**,**e**) and Cu_2_O (**c**,**f**) calculated at the PBE+U level of theory. The pDOS of the left panel refer to pristine bulk structures while those of the right panel pertain to Cu-defective structures.

The electronic features of both the pristine and the Cu-defective bulk structures have been analyzed in terms of the atom- and orbital momentum-projected density of states (*p*DOS) shown in Figure 2. CuI and Cu_2_O are both direct bandgap semiconductors with transitions occurring in the Ґ point of Brillouin zone, with experimental bandgaps of 3.1 eV [56] and 2.17 eV [57], respectively, while CuSCN presents an indirect Ґ—K bandgap of 3.60 eV [54]. Our calculations properly reproduced the semiconductive character for all three solids with the expected underestimation error for the bandgap values. Indeed, we predicted a bandgap of 2.1 eV for γ-CuI, in agreement with other theoretical calculations at both GGA+U (1.89 eV) [31] and HSE06 levels of theory (2.59 eV) [58] and a bandgap of 2.52 eV for β-CuSCN, also in agreement with previous theoretical works (2.1 eV) [59]. The well-known limitations of DFT in predicting bandgaps is most evident in the case of Cu_2_O, [60,61] and also our computed value (0.74 eV) is farther from the experimental one than for CuI and CuSCN.

From an electronic perspective, the presence of the Cu vacancy defect modifies their main features, as evidenced from Figure 2. In all three cases, the Fermi level crosses the valence band, hence confirming that Cu defects can be responsible for the experimentally observed p-type character. In CuI and Cu_2_O, both iodide and oxygen, along with the copper 3D orbitals, contribute to the emergence of acceptor states while in CuSCN. Besides copper, a major role is played by the thiocyanate ion.

We have modeled stable surfaces of each HTL with slab models carved from the optimized bulk structures. In this case, the presence of native defects has been represented by removing a copper atom from the central (inner) layer of each slab.

**Figure 3:**
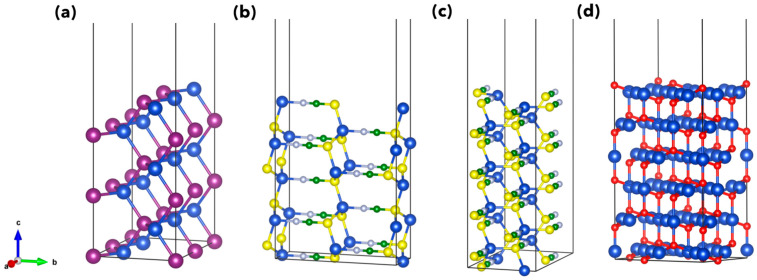
Slab models of (110) CuI surface (**a**), (100) and (110) CuSCN surfaces (**b**,**c**) and $\sqrt{3}\times \sqrt{3}R\left(30{}^{\circ}\right)$ (111) Cu_2_O surface (**d**). All slabs are shown before atomic relaxation. Color label for atomic spheres: Cu, dark blue; I, violet; S, yellow; C, green; N, light blue; O, red.

For CuI, we modeled the (110) surface, being one of the highest peaks in X-ray measurements [62], from a six-layer slab of the 2 × 1 × 1 bulk supercell (for a total of 48 atoms). The most stable surfaces of CuSCN are the polar (100) and the non-polar (110) [30], modeled here with nine-layer slabs. The (100) surface has been cleaved from a 4 × 1 × 1 supercell, while for the (110), we considered the 1 × 2 × 1 one (144 atoms in both cases). For Cu_2_O, we considered the most stable (111) surface [63]. Although still under debate [47], we considered a $\sqrt{3}\times \sqrt{3}R\left(30{}^{\circ}\right)$ supercell in order to reproduce the experimentally observed reconstruction phenomenon with this periodicity due to the loss of an oxygen atom from the outer surface layer [64,65,66]. Our Cu_2_O (111) slab consists of six atomic layers, with both a copper atom removed from the central inner layer and an oxygen atom missing in the surface. All surface models described above are shown in Figure 3.

**Figure 4:**
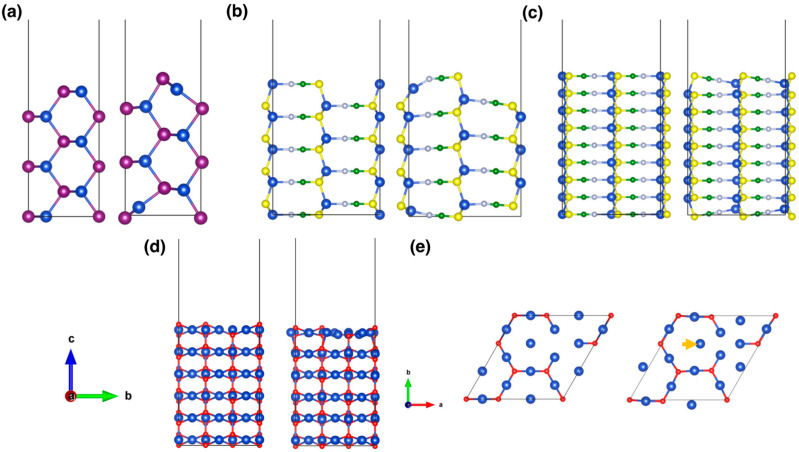
Side views of the pristine slab models shown before (left) and after (right) the structural relaxation for the (110) CuI slab (**a**), the (100) and the (110) CuSCN slabs (**b**,**c**) and the $\sqrt{3}\times \sqrt{3}R\left(30{}^{\circ}\right)$ (111) Cu_2_O surface (**d**). Top view of the topmost layer of Cu_2_O slab before (left) and after (right) relaxation (**e**). None of the structures present Cu vacancies in the atomic arrangement.

Even though to a different extent, all surfaces undergo structural reorganization upon optimization, as shown in Figure 4. Both CuI and CuSCN present a qualitatively similar surface reconstruction, where copper atoms tilt inwards and the anions (iodine/thyocianate moieties) are exposed towards vacuum, with this effect being more pronounced in CuI. In both cases, such structural changes involve mainly the surface layer and propagate only slightly to sub-surface and inner layers. Regarding Cu_2_O, our calculations predict only a slight in-plane surface reconstruction of the surface where copper atoms move towards the oxygen vacancy.

**Figure 5:**
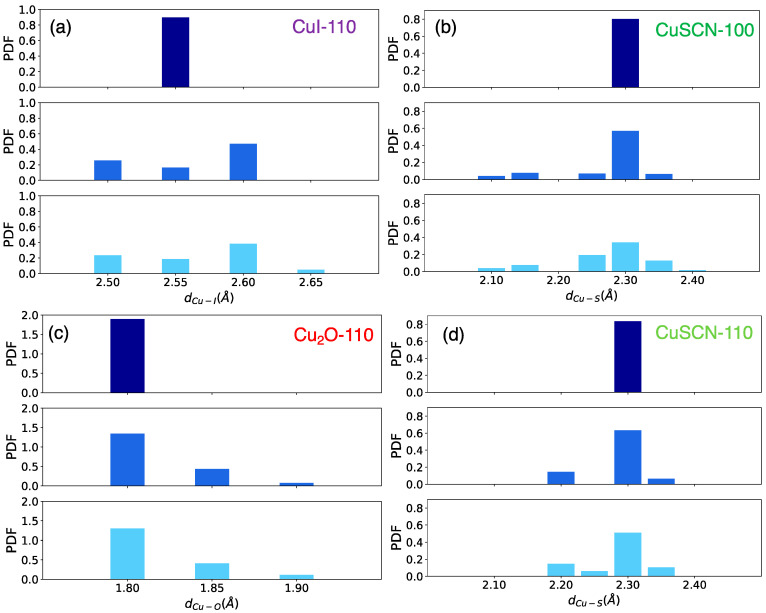
Pair distribution functions of: (**a**) Cu-I bond distances for the CuI slab, (**b**,**d**) for the Cu-S bond distances of the (100) and the (110) CuSCN slabs, respectively, and (**c**) Cu-O bond distances for the Cu_2_O slab. Dark blue color is used for structures before relaxation, while medium and light blue refer to the pristine and the defective optimized structures, respectively.

The three materials also show a different response upon the formation of the Cu defect from both structural and electronic points of view. Structural changes have been tracked by means of pair distribution functions (PDF) of the Cu-anion distances (Figure 5). From the PDF of CuI (Figure 5a), it is evidenced that the presence of the vacancy only slightly modifies the structure of the pristine CuI slab. Some of the Cu-I bond lengths become greater than 2.60 Å, the highest distance found in the pristine structure. These increased distances are localized in the proximity of the vacant site, which determines a further relaxation of the structure.

Regarding the (100) and the (110) CuSCN surfaces, the removal of a copper atom has a small elongation effect on Cu-S bond lengths next to the vacancy of the first surface (Figure 5b), while a local shrinking of Cu-S distances is observed in the (110) surface, (Figure 5d).

Eventually, the Cu_2_O structure is the most insensitive to the defect formation, as evident from a comparison between the Cu-O distance distributions before and after the formation of vacancy (Figure 5c). PDFs pertaining to the other atom couples of CuSCN, for which no significant changes were observed, are reported for completeness in Figure S3 of the SI.

The electronic response to the formation of the defect has been investigated carrying out a Bader charge analysis (using the Henkelmann’s group program [67]). Computed electron increase/decrease on each atomic species of each HTM candidate upon formation of the Cu vacancy are reported in Table 1.

In all cases, Bader charge analysis rules out a localized effect leading to Cu^2+^ formation. In both CuI and Cu_2_O, the hole delocalizes on both Cu and anion sublattices. In the case of CuSCN (100) surface, both sulfur and nitrogen sublattices host the electronic hole, while in the (110) slab, a major role is played by the copper and sulfur atoms.

**Figure 6:**
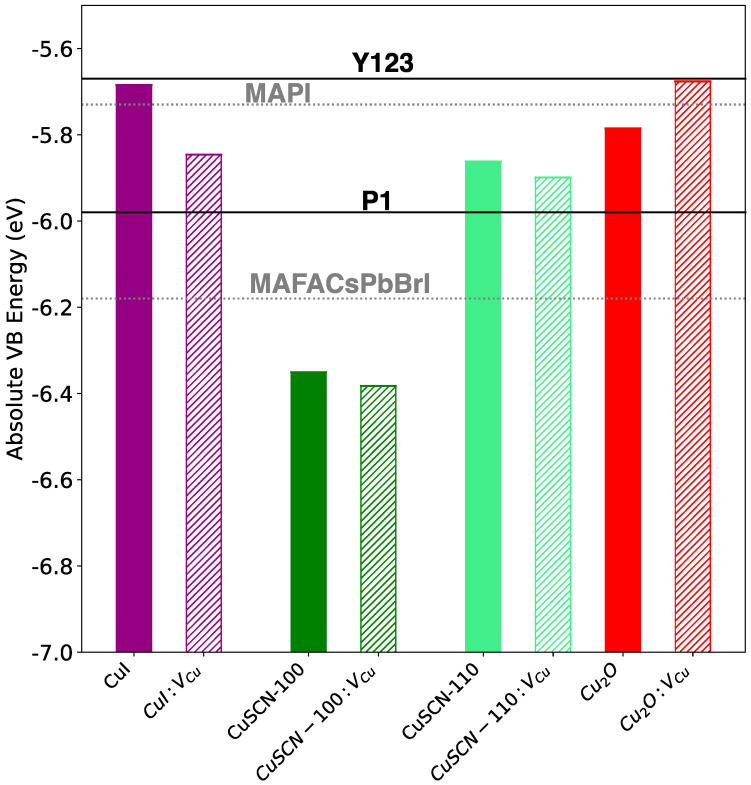
Absolute positions of the VB edges of pristine (fully colored rectangles) and Cu-defective (hatched rectangles) CuI, CuSCN and Cu_2_O slabs, in comparison to that of MAPI and triple cation MAFACsPbBrI perovskites (dotted gray lines) and the P1 and Y123 dyes’ HOMO (solid black lines).

For all the slab models, we evaluated the absolute positions of the valence band maximum, both for the pristine and the copper defective materials. For this purpose, we followed the approach proposed by Toroker et al. [35], already applied in previous literature to assess the driving force for hole or electron injection in heterogenous devices for PV applications [22,68]. Such an approach relies on the ability of DFT calculations to correctly predict bandgap center (BGC) positions, despite the failure in estimating quasi-particle excitations. In particular, Toroker et al. pointed out that the BGC positions are accurately predicted independently from the level of theory used in DFT calculations, and consequently even the so-computed absolute VBM positions are not significantly affected by this choice. In this framework, we employed the BGC positions of our slab models as predicted by DFT together with the experimental estimates of the bandgap for all the considered compounds CuI (3.1 eV) [56] CuSCN (3.60 eV) [54] and Cu_2_O (2.17 eV) [57]. Given that the bandgap center is ill-defined for non-stoichiometric systems, VB positions of p-type materials were shifted with respect to the pristine system according to the shift in the computed work functions. The calculated VB positions are reported in Figure 6 in comparison to that of MAPI, calculated within the same theoretical framework [68], the triple cation (MAFACsPbBrI) [69] and to the experimentally determined HOMO of the widely employed P1 [36] and Y123 [70] dyes.

These results reveal different behaviors for the considered materials. Notably, the VBMs of both CuSCN surfaces seem quite insensitive to the defect. Variations on the WFs are indeed of the order of meV, hence the valence band edges are virtually unmodified in both cases. This means a likely favorable match of the (110) surface with both the P1 dye’s HOMO and the triple cation perovskite, regardless from the presence of the defect.

On the contrary, the alignment with the other two photo-active systems, namely the MAPI and the Y123 dye, seems to prevent the charge transport for both surfaces.

Opposite trends were observed for the CuI and Cu_2_O. In the first case, the vacancy lowers the VBM, which hinders the charge transport at the MAPI junction while still preserving the proper conditions for the charge transfer with the P1 dye and the triple cation perovskite. In the Cu_2_O case, the defect pushes upwards the valence band edge and is crucial to ensure the proper alignment with all the considered absorbers.

A possible reason for the CuI behavior can be found in the significant structural reconstruction it experiences. The tilting of Cu-I bond axes over the whole structure can be responsible for the emergence of an electric dipole confining electrons. Such a dipole increases after removal of the copper atom due to a further localized relaxation of the structure next to the defect that stretches the distance between the copper atom and the I anion.

## 4. Conclusions

In this work, we have performed a comparative theoretical study on CuI, CuSCN and Cu_2_O, focusing on the role of copper defects on the band alignment and its implications in PV devices

We first investigated the three bulk phases both in the pristine and the Cu-defective cases and our results are in line with current literature.

We calculated, with a consistent approach, the VBM edge potential of each material within a theoretical framework that involves the slab models, for which we considered the most stable exposed facets. We found that in CuI, the presence of a defect lowers the VBM, likely due to a peculiar structural reconstruction in which the tilting of Cu-I bond axes generates dipole moments that are increased by a further localized reconstruction driven by the defect. Such a reduction of the VBM leads to an unfavorable band alignment with respect to the MAPI perovskite VBM, while preserving a good match with the triple cation and the P1 dye’s HOMO. In the thiocyanate, the copper vacancy slightly modifies the structure but has no visible effects on the VBM position. Our model predicts a likely good alignment of the (110) surface with the P1 dye and the triple cation perovskite while none of the two surfaces seem suitable for applications in devices involving both MAPI and Y123.

In a similar way, the presence of the vacancy does not modify the Cu_2_O structure. However, in this case, the defect is crucial to allow for the charge transfer across the interface with all photo-absorbers.

In conclusion, our calculations predict CuI to be a suitable p-type material for DSCs with P1 dye and PSCs involving MAFACsBrI, but also suggest controlling the presence of vacancies. Copper vacancies indeed play a beneficial role in enhancing the concentration of p-type carriers, but can jeopardize the charge transport across interfaces, as we found in the case of MAPI. On the contrary, copper oxide is a suitable photocathode in a Y123-/P1-based DSC and triple cation-based PSCs, while the increasing of VBM in p-Cu_2_O also makes it a good choice in the case of devices involving MAPI. It is, however, worth noting that a too high energy difference, i.e., a too high position of the photoanode/HTL valence band edge, can worsen the PCEs as it decreases the open circuit voltage.

This work compared the three p-type semiconductors and provides new insights into the effect of copper vacancies and their implications on the application of these materials as photoanodes/HTLs. These results are a probe of the possible charge transport effectiveness at interfaces. Our ongoing and future work will build upon these results to further characterize the HTM interfaces with photoactive molecules (for DSC) and perovskite-based materials (for PSC). Future complementary investigations, including surface defects and the effects of intrinsic doping on carrier mobility, could enrich the understanding of these compounds and provide further insights for interface engineering.

## Figures and Tables

**Table 1:** Change in the Bader charges (**Δq** in e^−^) upon formation of the copper vacancy in CuI, CuSCN and Cu_2_O surfaces. All entries indicate the total cumulative charge change in the corresponding sublattice.

Dq(e^−^)	Cu	I/O	SCN	S	C	N
**CuI**	−0.11	−0.17		-	-	-
**CuSCN-100**	−0.06	-	−0.47	−0.35	0.26	−0.38
**CuSCN-110**	−0.29	-	−0.21	−0.49	0.11	0.17
**Cu_2_O**	−0.33	−0.20		-	-	-

## Supplementary Materials

The following supporting information can be downloaded at: https://www.mdpi.com/article/10.3390/ma15165703/s1. Figure S1: Schematic chemical structures of Spiro-OMeTAD, PEDOT:PSS, PTAA and P1 and Y123 dyes mentioned in this work. Figure S2: Bulk models shown before relaxation of (a) unit cell of cubic CuI, (b) unit cell hexagonal CuSCN and (c) unit cell of cubic Cu_2_O. Color label for atomic spheres: Cu—dark blue; I—violet; S—yellow; C—brown; N—light blue; O—red. Figure S3: Pair distribution functions of, from left to right: Cu-N, C-N and C-S distances for the CuSCN-100 surface slabs (upper panel ) and CuSCN-110 surface slabs (lower panel). Dark blue color is used for structures before relaxation while medium and light blue refer to the pristine and the defective optimized structures, respectively. Table S1: Lattice constants of CuI, CuSCN and Cu_2_O calculated within the PBE+U level of theory. Experimental values are reported for comparison in the last column. Reference [71] is cited in the supplementary materials.
