# Supplementary material for: First-Principles Study of Cu-Based Inorganic Hole Transport Materials for Solar Cell Applications
Source: Materials (Basel). 2022 Aug 18;15(16):5703. doi: 10.3390/ma15165703 (PMC9413571; doi:10.3390/ma15165703)
Supplement: Supplementary file 1 [file materials-15-05703-s001.zip › materials-1819322-supplementary.pdf]

Supporting Information to

First principles study of Cu-based inorganic hole transport materials for application in solar cells

Adriana Pecoraro, [a] Pasqualino Maddalena, [a] Michele Pavone[b] and Ana B. Muñoz García, *[a]

[a]*Department of Physics "Ettore Pancini", University of Naples Federico II*
[b]*Department of Chemical Sciences, University of Naples Federico II*

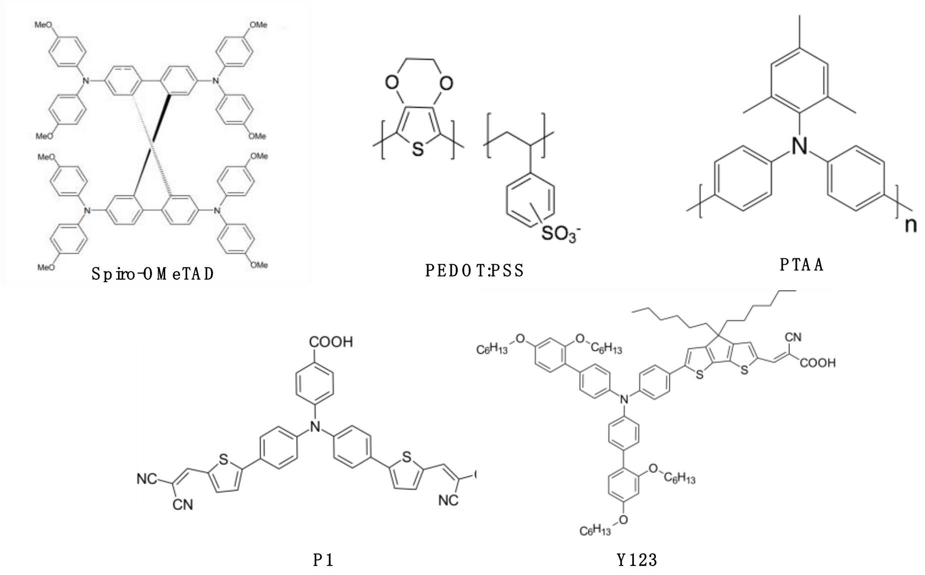

Figure S1: Schematic chemical structures of Spiro-OMeTAD, PEDOT:PSS, PTAA and P1 and Y123 dyes mentioned in this work.

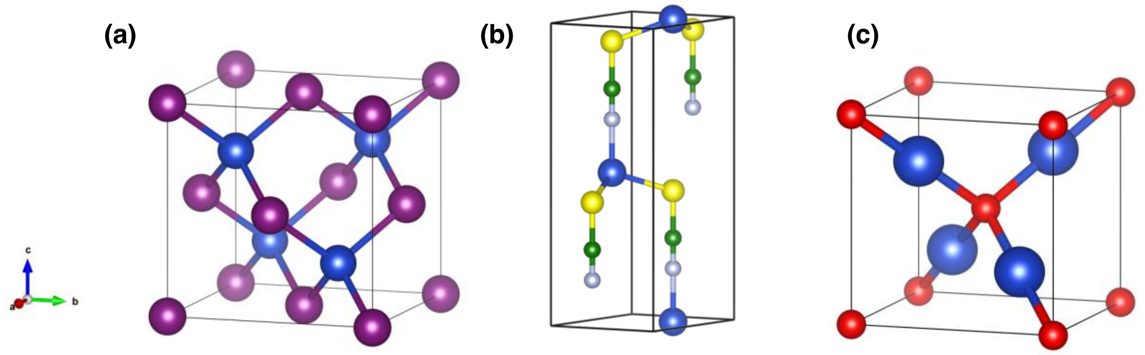

Figure S2: Bulk models shown before relaxation of (a) unit cell of cubic CuI, (b) unit cell hexagonal CuSCN and (c) unit cell of cubic Cu_2O. Color label for atomic spheres: Cu - dark blue; I – violet; S – yellow; C – brown; N – light blue; O – red.

Table S1: Lattice constants of CuI, CuSCN and Cu_2O calculated within the PBE+U level of theory. Experimental values are reported for comparison in the last column.

	**Lattice constant (Å)**	**This work**	**Exp**
**CuI**	*a*	6.003	6.058[1]
**CuSCN**	*a*	3.831	3.850
	c	10.883	10.938[2]
**Cu_2O**	*a*	4.234	4.270[3]

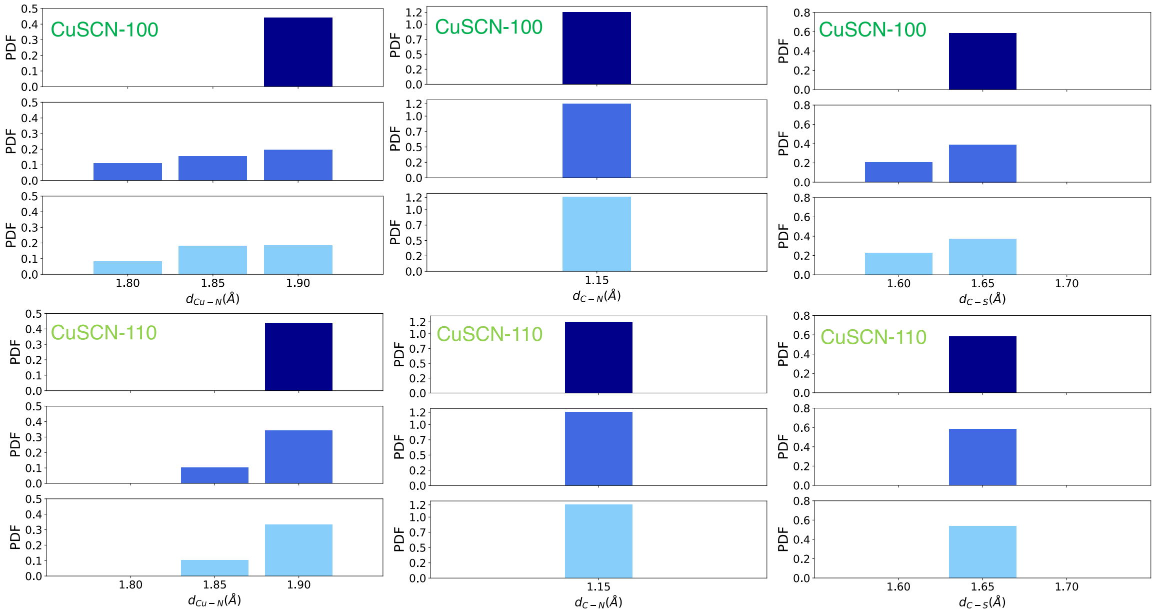

Figure S3: Pair distribution functions of, from left to right: Cu-N, C-N and C-S distances for the CuSCN-100 surface slabs (upper panel) and CuSCN-110 surface slabs (lower panel). Dark blue color is used for structures before relaxation while medium and light blue refer to the pristine and the defective optimized structures, respectively.

References

[1] M. Yashima, Q. Xu, A. Yoshiasa, and S. Wada, J. Mater. Chem. **16**, 4393 (2006).
[2] D.L. Smith and V.I. Saunders, Acta Cryst B **38**, 907 (1982).
[3] A. Sanson, F. Rocca, G. Dalba, P. Fornasini, R. Grisenti, M. Dapiaggi, and G. Artioli, Phys. Rev. B **73**, 214305 (2006).
